# Nutritional composition, antioxidant and antimicrobial properties of *Opuntia ficus-indica* (L) Miller cladodes and fruits

**DOI:** 10.1007/s11130-026-01478-0

**Published:** 2026-03-02

**Authors:** Itana Vivian Rocha Santana, Giovanni Eiji do Nascimento Ozaki, Fabricio Luiz Tulini

**Affiliations:** Federal University of Western Bahia, Barreiras, Brazil

**Keywords:** Cactaceae, Functional foods, Prickly pear, Proximal composition

## Abstract

*Opuntia ficus-indica* (L.) Miller is an arid-adapted species native to Mexico that is rich in dietary fiber and bioactive compounds, making it valuable for applications in food, medicine, and animal feed. Thus, this study aimed to analyze the nutritional and bioactive properties of cladodes and fruits from this species. Proximate composition analysis revealed that both cladodes and fruits have high moisture content and low lipid and carbohydrate levels. They also provide dietary fiber (4.470 to 7.720 g/100 g), protein (0.623 to 1.033 g/100 g), and minerals (0.893 to 0.840 g/100 g), thereby enhancing their nutritional profile. Antimicrobial assays demonstrated that extracts from fruits and cladodes possess significant inhibitory activity against Gram-positive and Gram-negative bacteria, yeasts, and molds. Notably, the cladode extract demonstrated strong activity against *Staphylococcus aureus*, suggesting potential for use in food preservation and therapeutic applications after additional studies. Phytochemical analyses confirmed the presence of coumarins, steroids, and terpenoids in both cladode and fruit extracts, whereas tannins were not detected. Antioxidant activity showed that cladode extracts have significantly higher antioxidant potential than fruit extracts, particularly in total phenolic content (11.919 mg Gallic acid equivalents/g) and ferric reducing capacity (1.561 µmol Trolox equivalents/g), suggesting that cladodes may serve as a source of natural antioxidants. Overall, these findings suggest that *O. ficus-indica* cladodes and fruits may be promising sources of bioactive compounds for functional food applications, highlighting the importance of further research to better characterize their composition and associated properties.

**Supplementary Information:**

The online version contains supplementary material available at 10.1007/s11130-026-01478-0.

## Introduction

 Approximately 31% of cacti worldwide are threatened by factors like land use changes, invasive species, overharvesting, and climate change, which highlights the urgent need for conservation efforts targeting this plant family [1, 2]. *Opuntia ficus-indica (L)* Miller (Cactaceae), commonly known as the prickly pear cacti, is a species native to Mexico and widely distributed throughout other Latin American countries, the Mediterranean region, and South Africa, and it is well adapted to difficult environmental conditions [3–5]. *O. ficus-indica* cladodes are rich in dietary fiber (57% on a dry-weight basis), and they are used as animal feed, culinary ingredients in various dishes, and in traditional medicine to treat bruises, burns, wounds, and infections [6, 7]. Additionally, *O. ficus-indica* fruits are used in food processing to produce juices, preserves, fermented beverages, flours, natural dyes, and other value-added products [6].

Overproduction of reactive oxygen species (ROS) can result in cellular damage, especially during aging or poor health, and this damage is associated with various diseases such as inflammation, arteriosclerosis, arthritis, cancer, and the natural aging process. Therefore, to promote health, it is vital to consume foods or substances that can effectively neutralize free radicals [8]. While antioxidants are essential for preventing oxidative stress, some synthetic variants may pose health risks, highlighting the need for safer and natural alternatives. Plant-derived compounds represent a promising alternative for combating free radicals and supporting treatments for diseases associated with oxidative stress [9]. In this regard, Cactaceae species produce a variety of beneficial compounds, including glycosylated flavonols, dihydroflavonols, flavanones, flavonols, betaxanthins, betacyanins, and other phenolic compounds, all of which are recognized for their antioxidant properties. The composition and concentration of these compounds can vary depending on the specific plant tissue, variety, and ripening stage [10]. Certain phenolic compounds, such as quercetin, isorhamnetin, and kaempferol, have also shown significant antimicrobial activity against a range of pathogens, including *Bacillus subtilis*, *Escherichia coli*, *Pseudomonas aeruginosa*, and *Klebsiella pneumoniae* [2]. As antimicrobial resistance increases and microbial sources for antibiotics decline, the search for novel plant-based antimicrobials has become increasingly urgent, particularly from *Opuntia* species.

Despite its adaptability to semi-arid regions and the recognized nutritional and bioactive potential, *O. ficus-indica* remains underutilized in human nutrition. Therefore, this study aimed to characterize the proximate composition and evaluate the antioxidant and antimicrobial properties of *O. ficus-indica* cladodes and fruits, with a focus on their potential applications in human nutrition and functional foods.

## Materials and methods

The materials and methods section is provided as an Online Resource (supplementary material).

## Results and discussion

The proximate composition of *O. ficus-indica* fruits and cladodes was evaluated, and the results are presented in Table 1.

**Table 1:** Proximate composition of *Opuntia ficus-indica* fruits and cladodes, expressed as mean ± standard deviation. The caloric values were calculated using 4.0 kcal/g for proteins, 9.0 kcal/g for lipids and 4.0 kcal/g for carbohydrates

	Cladodes	Fruits
Moisture (g/100 g)	91.700 ± 0.776	82.760 ± 0.404
Ash (g/100 g)	0.893 ± 0.040	0.840 ± 0.078
Lipids (g/100 g)	0.010 ± 0.000	0.026 ± 0.020
Dietary fibers (g/100 g)	4.470 ± 0.474	7.720 ± 0.422
Proteins (g/100 g)	0.623 ± 0010	1.033 ± 0.055
Carbohydrates (g/100 g)	2.304	7.621
Caloric value (kcal/100 g)	11.717	31.131

Both fruits and cladodes exhibited high moisture content and low lipid levels, along with significant amounts of ash, protein, and dietary fiber. Consequently, both are considered low in caloric value, highlighting their potential for use in the production of health products. In Mexico, Uriarte-Frías et al. [11] produced flours from dried cultivated *O. ficus-indica* cladodes and assessed their proximate composition, reporting higher protein and ash contents than those observed in the present study. Likewise, Guevara-Figueroa et al. [12], when evaluating the proximate composition of commercial and wild *O. ficus-indica* cladodes, also reported higher protein and ash values than those in the present study. Additionally, when examining wild *O. ficus-indica* fruits in Ethiopia, Yiblet et al. [13] observed lower levels of ash, dietary fiber, and carbohydrates, but higher concentrations of proteins and lipids. The nutritional profile of *O. ficus-indica* differs among species, varieties, or plant parts, and it is strongly influenced by environmental conditions, agronomic practices, and post-harvest processing, which collectively affect its physicochemical and nutritional properties [6]. The chemical and mineral compositions of *O. ficus-indica* cladodes also change with maturity, as older cladodes are more abundant in carbohydrates and minerals, whereas younger cladodes contain higher protein levels [14].

The phytochemical analyses revealed the presence of coumarins, steroids, and terpenoids in both extracts, while fatty acids and tannins were not detected, as shown in Table 2.

**Table 2:** Results of the phytochemical analyses of the hydroalcoholic extracts of *Opuntia ficus-indica* cladodes and fruits

	Cladode	Fruit
Coumarins	positive	positive
Fatty acids	negative	negative
Steroids	positive	positive
Tannins	negative	negative
Terpenoids	positive	positive

Although the occurrence of coumarins in *Opuntia* species remains largely unexplored, Mohammed et al. [15] identified daphnetin and 4-methylcoumarin in *O. ficus-indica* fruit extracts using UPLC-MS/MS analyses. Coumarins are widely found in plants, bacteria, and fungi, and they are characterized by a structure that comprises a fused benzene and pyrone ring, belonging to the category of low-molecular-weight phenolics [16]. Based on their chemical structure, coumarins can be categorized into several types, including simple coumarins (or hydroxycoumarins), simple prenylated and geranylated coumarins, furanocoumarins, pyranocoumarins, sesquiterpenyl coumarins, and oligomeric coumarins [17]. In terms of biological activities, coumarins are recognized for their anticoagulant, antioxidant, antiangiogenic, anticancer, and antimicrobial properties [16].

Steroids are a class of compounds characterized by their steroid nucleus, which is formed from a phenanthrene ring structure fused with a pentane ring. When this structure is fully saturated with hydrogen, it forms what is known as a sterane ring structure [18]. Plant steroids exhibit a wide range of beneficial properties, making them valuable in both pharmaceutical and agricultural products. These include anti-tumor, immunosuppressive, hepatoprotective, and antibacterial activities, as well as functions as plant growth regulators, anthelmintic agents, and cytotoxic and cardiotonic effects [19]. Another class of bioactive compounds identified in the present study is the terpenoids, which are divided into monoterpenes, sesquiterpenes, and diterpenes. These compounds play essential roles in plant interactions, attracting pollinators and seed dispersers while defending against insects and pathogens. They contribute to photosynthesis and respiration, regulate growth through hormones, modulate membrane fluidity, and provide photoprotection [20]. Sánchez et al. [21] reported the presence of terpenoids, tannins, and coumarins in *O. ficus-indica* cladode extracts, although steroids were not detected in their study. With regard to *O. monacantha* fruit extracts, Silva et al. [22] identified the terpenes *α*-amyrin and *β*-amyrin, although they used hexane as a solvent for extraction. Variations in extraction methods and analytical procedures may account for differences in the phytochemical profiles reported across studies.

Antioxidants can be categorized into two main groups based on their reaction mechanisms, either by inhibiting oxidant enzymes or by directly reacting with reactive oxygen/nitrogen species (ROS/RNS), producing less toxic or reactive products [23]. To assess antioxidant activity, chemical-based assays are the most commonly used and can be categorized into three types: those that measure the scavenging activity of stable free radicals (DPPH and ABTS), those that assess metal ion reduction (FRAP, CUPRAC, and the Folin-Ciocalteu assay), and those that use competitive methods (ORAC and TRAP) [24]. When selecting an antioxidant assay, it is essential to consider the polarity and solubility of the antioxidant compounds. The DPPH assay is suitable for evaluating hydrophobic compounds, FRAP and ABTS are commonly used for hydrophilic compounds, and ABTS has the advantage of assessing both hydrophobic and hydrophilic compounds [24]. Given these characteristics, using at least two different assays is crucial for an accurate evaluation of the antioxidant activity of natural products.

**Fig. 1:**
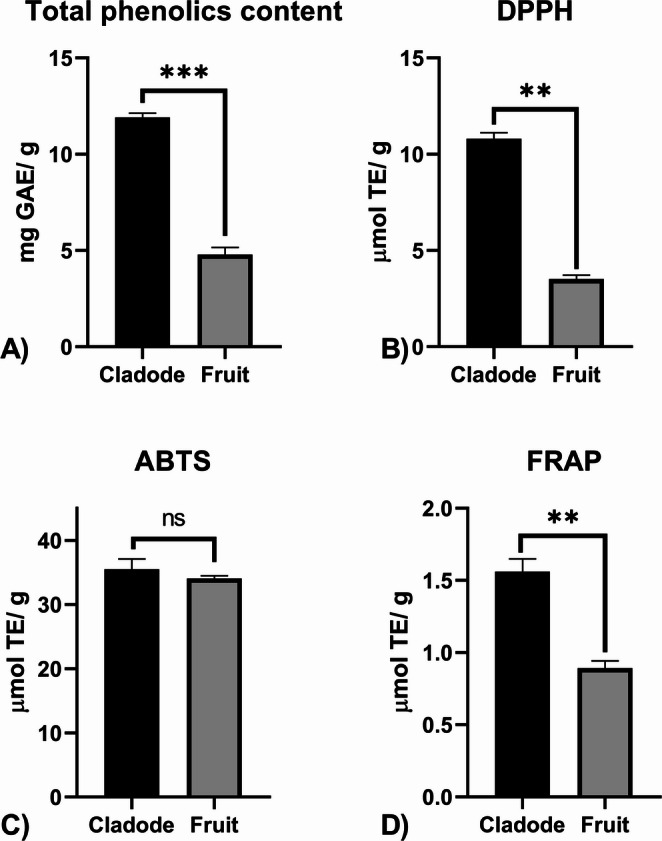
Total phenolic content (**A**), expressed as milligrams of gallic acid equivalents per gram of dry weight, and antioxidant activity assessed using the DPPH (**B**), ABTS (**C**), and FRAP (**D**) methods, expressed as micromoles of Trolox equivalents per gram of dry weight, for the hydroalcoholic extracts of *Opuntia ficus-indica* cladodes and fruits. Significant differences are indicated by * (*p* < 0.05), ** (*p* < 0.01), and *** (*p* < 0.001)

Quantification of total phenolics showed that the cladode extract contains higher phenolic levels than the fruit extract (Fig. 1). A similar pattern in antioxidant potential was observed in the DPPH and FRAP assays, with the cladode extract showing stronger antioxidant activity. However, in the ABTS assay, both extracts showed similar antioxidant activity. Andreu et al. [25] reported similar phenolic levels in extracts from cladodes and fruits of *O. ficus-indica* (15.6 and 13.0 mg GAE/g, respectively). However, they observed higher antioxidant activity in fruit extracts than in cladode extracts using ABTS and DPPH assays, whereas the FRAP assay showed greater antioxidant activity in cladode extracts. Additionally, they reported a reduction in antioxidant activity when older cladodes were analyzed. It is important to note that they used acidified methanol instead of ethanol for extraction, which may have influenced the bioactive compound profile and introduced limitations when comparing their results with those of the present study. In addition, as previously discussed, the DPPH assay primarily evaluates hydrophobic antioxidants, whereas the FRAP assay targets hydrophilic compounds, and the ABTS assay assesses both hydrophilic and hydrophobic antioxidants [24].

A positive correlation between FRAP and ABTS results was also reported by Wootton-Beard et al. [26] when evaluating vegetable juices, while a low correlation was observed with DPPH results. According to López-Alarcón et al. [23] the diversity of compounds in plants can lead to varying responses in antioxidant assays, depending on the phenolic profile. This variation can be explained by the synergistic activity of certain compounds on specific antioxidant mechanisms or by the higher metal-reducing potential of some phenolic compounds.

The results of the antimicrobial analyses are presented in Table 3. Both cladode and fruit extracts showed broad-spectrum inhibitory activity, effectively targeting Gram-positive and Gram-negative bacteria, as well as yeast and molds.

**Table 3:** Inhibition halo diameters (mm) of *Opuntia ficus-indica* extracts obtained from cladodes and fruits. A 60% (v/v) aqueous ethanol solution was used as the negative control. Different letters whitin a row indicate significant differences according to one-way ANOVA (analysis of variance) followed by Tukey’s test (*p* < 0.05)

**Gram-positive bacteria**	**Negative control**	**Cladode**	**Fruit**
*Bacillus cereus* FT10	5.00 ± 0.00 ^a^	12.67 ± 0.58 ^b^	9.67 ± 0.58 ^c^
*Enterococcus faecalis* ATCC 29212	6.00 ± 0.00 ^a^	8.00 ± 0.00 ^b^	9.00 ± 0.00 ^b^
*Enterococcus faecium* EM03	5.00 ± 0.00 ^a^	10.33 ± 0.58 ^b^	10.67 ± 0.58 ^b^
*Listeria monocytogenes* DSM 19094	6.00 ± 0.00 ^a^	13.67 ± 0.58 ^b^	12.33 ± 0.58^c^
*Pediococcus acidilactici* LK08	6.00 ± 0.00 ^a^	6.00 ± 0.00 ^a^	6.00 ± 0.00 ^a^
*Staphylococcus aureus* WDCM 00212	7.00 ± 0.00 ^a^	20.33 ± 1.15 ^b^	7.00 ± 0.00 ^a^
**Gram-negative bacteria**	**Negative control**	**Cladode**	**Fruit**
*Escherichia coli* ATCC 29212	7.67 ± 0.58 ^a^	6.00 ± 0.00 ^b^	14.00 ± 0.00 ^c^
*Klebsiella pneumoniae* ATCC 700603	7.00 ± 0.00 ^a^	13.00 ± 1.00 ^b^	15.00 ± 1.00 ^c^
*Pseudomonas aeruginosa* ATCC 27853	7.00 ± 0.00 ^a^	10.33 ± 0.58 ^b^	14.00 ± 0.00 ^c^
*Salmonella* Enteritidis NCTC 6676	6.67 ± 0.58 ^a^	14.00 ± 0.00 ^b^	15.00 ± 1.00 ^b^
**Yeast and molds**	**Negative control**	**Cladode**	**Fruit**
*Aspergillus brasiliensis* ATCC16404	5.00 ± 0.00 ^a^	10.15 ± 0.21 ^b^	11.15 ± 0.21 ^b^
*Botrytis cinerea* FAT1252	5.00 ± 0.00 ^a^	9.10 ± 0.14 ^b^	8.10 ± 0.14 ^b^
*Candida albicans* ATCC 10231	7.00 ± 0.00 ^a^	11.33 ± 0.58 ^b^	13.00 ± 1.00 ^c^
*Penicillium roqueforti* ATCC 10110	5.00 ± 0.00 ^a^	11.05 ± 0.07 ^b^	8.10 ± 0.14 ^c^
*Trichoderma reesei* ATCC 26921	5.00 ± 0.00 ^a^	12.10 ± 0.14 ^b^	16.15 ± 0.21^c^

Both extracts exhibited significant inhibitory activity against *B. cereus*, *E. faecalis*, *E. faecium*, and *L. monocytogenes*, but only the cladode extract was effective against *S. aureus*, highlighting its potential against this highly pathogenic strain. Overall, the strong inhibitory effects observed against these pathogens reinforce the potential of *O. ficus-indica* extracts as effective antimicrobial agents. Interestingly, the lactic acid bacterium (LAB) *P. acidilactici* was not inhibited by the extracts, which represents a desirable characteristic for food biocontrol applications. This selective antimicrobial activity suggests that these extracts may effectively suppress pathogenic bacteria while preserving beneficial lactic acid bacteria and/or probiotic microorganisms. With regard to Gram-negative bacteria, the fruit extract inhibited all tested strains, including *E. coli*, *K. pneumoniae*, *P. aeruginosa*, *S. Enteritidis*, as well as the yeast *C. albicans*. In contrast, the cladode extract was ineffective against *E. coli*, illustrating the differences in antimicrobial efficacy between the two extracts. When tested against mold strains, both extracts inhibited the growth of *(A) brasiliensis*, *(B) cinerea*, *P. roqueforti* and *T. reesei*. Despite differences in extract production, microorganism strains, age of the cladodes, seasonal temperatures, rainfall, soil type, and general growing conditions, other researchers have reported the antimicrobial activities of *Opuntia* extracts against Gram-positive, Gram-negative, and fungal species [27–29]. In a different approach, Benramdane et al. [30] extracted lipophilic compounds from the roots of *O. ficus-indica* and observed significant inhibitory activity against *E. coli*, *P. aeruginosa*, *S. aureus*, and *B. cereus*. Similarly, Alqurashi et al. [31] evaluated the antimicrobial potential of the *O. ficus-indica* seed oil extract and reported significant inhibitory activity against *E. coli*, *K. pneumoniae*, *P. aeruginosa*, *S. aureus*, and *B. subtilis*, as well as against the yeast *Saccharomyces cerevisiae* and the fungus *P. digitatum*. These findings, together with the results of the present study, highlight the need for further investigations to better characterize the bioactive compounds involved in the antimicrobial activity of *O. ficus-indica* and to evaluate their biological relevance.

The inhibitory activity observed for the cladode extract against *S. aureus* provides preliminary evidence of antimicrobial potential under the experimental conditions evaluated in this study. Although these results demonstrate measurable biological activity, they should be interpreted as exploratory. Further investigations are required to identify the bioactive compounds responsible for this effect and to elucidate their significance beyond in vitro assays. Similarly, the inhibitory effects detected against mold strains suggest that the cladode extract contains compounds with antifungal potential. However, the practical relevance of these findings cannot be established based on the present data alone. Future studies will therefore be essential to evaluate the stability, safety, and efficacy of these bioactive compounds across diverse biological systems and environmental conditions.

## Conclusions

This study demonstrated that *O. ficus-indica* cladodes and fruits exhibit high moisture content and low lipid and carbohydrate levels, supporting their classification as low-calorie food resources. Phytochemical screening of the hydroalcoholic extracts revealed the presence of coumarins, steroids, and terpenoids, which were associated with notable antioxidant and antimicrobial activities. Both fruit and cladode extracts exhibited significant inhibitory activity against Gram-positive and Gram-negative bacteria, yeasts and molds, with the cladode extract showing activity against *S. aureus*. These findings emphasize the potential of *O. ficus-indica* cladodes and fruits as functional food ingredients and valuable natural sources of bioactive compounds. Nevertheless, further research is required to elucidate their detailed chemical composition and to support their safe, effective, and sustainable use in food and related applications.

## Supplementary Information

Below is the link to the electronic supplementary material.

Supplementary file 1 (DOCX 25.8 KB)

## Data Availability

No datasets were generated or analysed during the current study.
