# Supplementary material for: Nutritional composition, antioxidant and antimicrobial properties of Opuntia ficus-indica (L) Miller cladodes and fruits
Source: Plant Foods Hum Nutr. 2026 Mar 2;81(1):26. doi: 10.1007/s11130-026-01478-0 (PMC12953465; doi:10.1007/s11130-026-01478-0)
Supplement: Supplementary file 1 — (DOCX 25.8 KB) [file 11130_2026_1478_MOESM1_ESM.docx]

**SUPPLEMENTARY MATERIAL**

**Nutritional composition, antioxidant and antimicrobial properties of *Opuntia ficus-indica* (L) Miller cladodes and fruits**

Itana Vivian Rocha Santana^1^; Giovanni Eiji do Nascimento Ozaki^1^; Fabricio Luiz Tulini^1*^

^1^Federal University of Western Bahia, Brazil.

*corresponding author – [fabricio.tulini@ufob.edu.br](mailto:fabricio.tulini@ufob.edu.br)

**1. Materials and methods**

**1.1 Plant material**

The cladodes and fruits of *Opuntia ficus-indica* were randomly collected from naturally occurring populations in open natural areas within the municipality of Barra (11° 05′ 20″ S, 43° 08′ 31″ W) and Baianópolis (12° 18′ 21″ S, 44° 32′ 06″ W), respectively, in Bahia (Brazil). Upon collection, the samples spines were removed, followed by washing with water and detergent to eliminate impurities. Subsequently, the samples were sanitized with sodium hypochlorite and rinsed thoroughly under tap water before being air-dried at 25°C. These samples were then placed in plastic bags and frozen until the time of analysis.

**1.2. Reagents**

All commercial standards (Gallic acid and Trolox), as well as 2,2-Diphenyl-1-picrylhydrazyl (DPPH), 2,2'-azino-bis-(3-ethylbenzthiazoline-6-sulfonic acid) (ABTS) and 2,4,6-tri(2'-pyridyl)-1,3,5-triazine (TPTZ) were purchased from Sigma-Aldrich (St. Louis, USA). Folin-Ciocalteau reagent was purchased from Lioserum Chemicals (Ribeirão Preto, Brazil). All culture media and material for microbiology was purchased from Kasvi (Pinhais, Brazil).

**1.3 Proximal composition**

**1.3.1 Moisture Content**

Moisture content was evaluated by drying *ca.* 2 g of the samples at 105 °C in a laboratory stove until a constant weight was reached, calculating the mass lost during this process. The results were expressed as gram of moisture per 100 g of cladode or fruit [1].

**1.3.2 Ash Content**

The ash content in the samples was determined by burning *ca.* 2 g of the sample at 600 °C in a muffle until a constant weight was achieved. The results were expressed as gram of ash per 100 g of cladode or fruit [2].

**1.3.3 Lipids**

The lipid content was determined using a Soxhlet extractor. Approximately 5 grams of dried and powdered samples were placed in cartridges and inserted into the Soxhlet extractor system. The apparatus was filled with *ca.* 200 ml of petroleum ether, and the solvent circulated through the sample for 4 hours. Afterward, the solvent was evaporated, and the remaining material was weighed. The results were expressed as gram of lipids per 100 g of cladode or fruit [3].

**1.3.4 Proteins**

The protein content in the cladodes and fruits was assessed using the micro Kjeldahl method [4] with modifications. In this process, 4 ml of sulfuric acid were added to a glass tube containing *ca.* 0.2 g of the sample along with 2 g of copper sulfate and potassium sulfate in a 1:10 ratio. The mixture was heated to 360 °C until fully digested, after which nitrogen was distilled as ammonia using a Kjeldahl apparatus. The distilled ammonia was collected in 5 ml of a 2% (w/v) boric acid solution with methyl red, and the amount of nitrogen was quantified using a standard 0.1 M HCl solution. A correction factor of 6.25 was applied to calculate the protein content, with results expressed as gram of proteins per 100 g of cladode or fruit.

**1.3.5 Dietary Fiber**

The dietary fiber content in the cladodes and fruits was measured using the method of neutral detergent fiber (NDF) with modifications, focusing on analyzing cellulose, hemicellulose, and lignin [5]. For this procedure, *ca.* 0.5 g of the sample was combined with 5 ml of water and 0.2 ml of a thermostable amylase solution (Termamyl - Novozymes, Araucária, Brazil) and heated at 80 °C for 20 minutes. Subsequently, 35 ml of neutral detergent solution was added to the mixture, which was boiled for one hour. The dietary fibers were collected on pre-dried and tared quantitative filter paper and washed with 10 ml of acetone. After drying the filter with residues at 105 °C, the material was incinerated at 600 °C until a constant weight was reached to measure the ashes. The neutral detergent fiber content was calculated by determining the weight difference between the filter alone and the filter with residues (excluding ash weight), with results were expressed as gram of dietary fibers per 100 g of cladode or fruit.

**1.3.6 Carbohydrates**

The carbohydrate content was calculated by subtracting the percentages of moisture, ash, lipids, proteins, and dietary fibers from 100%, with results presented as gram of carbohydrates per 100 g of cladode or fruit.

**1.4 Extract preparation**

The cladodes and fruits were sliced and dried in an air-circulating stove at 55 °C for 72 hours. The dried material was then ground using a mortar and pestle until a fine powder was achieved. Next, *ca.* 1.25 g of fruit powder or 2.5 g of cladode powder were combined with a 60% (v/v) aqueous ethanol solution, resulting in a final volume of 25 ml. After 24 hours of agitation in a shaker set at 60 rpm, solid residues were removed through filtration, and the resulting extracts were stored at -20 °C until needed.

**1.5 Phytochemical analyses**

The hydroalcoholic extracts were analyzed to detect some classes of bioactive compounds [6]. Steroids were identified by dissolving 1 ml of extract in 10 ml of chloroform and adding an equal volume of concentrated sulfuric acid along the walls of the test tube. A red upper layer and a yellow lower layer with green fluorescence confirmed the presence of steroids. Terpenoids were detected by adding 2 ml of extract to 2 ml of acetic anhydride and 2 ml of concentrated sulfuric acid. The formation of blue or green rings indicated a positive result for terpenoids. Fatty acids were assessed by mixing 0.5 ml of extract with 5 ml of ether and allowing it to evaporate on filter paper. The appearance of transparency on the paper indicated the presence of fatty acids. Tannins were identified by adding 2 ml of extract to a few drops of 1% lead acetate. The formation of a yellow precipitate confirmed the presence of tannins. Finally, coumarins were detected by adding 3 ml of 10% (m/v) NaOH to 2 ml of extract. The formation of a yellow coloration confirmed their presence.

**1.6 Antioxidant activity**

**1.6.1 Total phenolic content**

The quantification of total phenolics was performed in the hydroalcoholic extracts, with some modifications [7]. In a 96-well microplate, 25 µl of Folin-Ciocalteu reagent was mixed with 25 µl of the extracts and 200 µl of distilled water. After 3 minutes, 25 µl of a 30% (m/v) aqueous sodium carbonate solution was added. The plate was then incubated for 30 minutes at 37 °C before absorbance was measured at 630 nm, and absolute ethanol was used as a blank control. A calibration curve was produced using gallic acid at concentrations of 40, 60, 80, 100, 120, 140, and 160 µg/ml. The results were expressed as milligrams of gallic acid equivalents (GAE) per gram of dry weight.

**1.6.2 DPPH (2,2-Diphenyl-1-picrylhydrazyl)** **radical scavenging assay**

The DPPH radical scavenging assay was conducted according to Mrkonjić et al. [8], with some modifications. A test tube was prepared by adding 2,900 µl of DPPH solution and 100 µl of the extracts, and this mixture was then left in the dark at room temperature for 1 hour, after which the absorbance was measured at a wavelength of 517 nm. The DPPH solution was prepared at a concentration of 26 µg/ml in ethanol, with the absorbance adjusted to 0.70 ± 0.04. A calibration curve was prepared using 2,900 µl of DPPH solution and 100 µl of Trolox at concentrations of 40, 60, 80, 100, 120, 140, and 160 µg/ml. The results were expressed as micromoles of Trolox equivalents (TE) per gram of dry weight.

**1.6.3 ABTS** (**2,2'-azino-bis-[3-ethylbenzthiazoline-6-sulfonic acid]) assay**

The ABTS assay was performed according to El Baakili et al [9], with some modifications. The ABTS working solution was prepared by combining 88 µl of a 140 mM potassium persulfate aqueous solution with 5,000 µl of a 7 mM ABTS aqueous stock solution. Prior to use, the mixture was stored in the dark for 16 hours at room temperature, and further diluted with ethanol to achieve an absorbance of 0.700 at a wavelength of 630 nm. In a 96-well microplate, 200 µl of the ABTS working solution and 15 µl of the extracts were added in each well, and the absorbance at 630 nm was measured after 6 minutes. A calibration curve was prepared using Trolox concentrations of 40, 60, 80, 100, 120, 140, and 160 µg/ml. The results were expressed as micromoles of Trolox equivalents (TE) per gram of dry weight.

**1.6.4 FRAP (Ferric Reducing Antioxidant Power) assay**

The FRAP assay was performed according to Yeerong et al. [10], with some modifications. The FRAP solution was prepared by combining 1.25 ml of a 10 mM TPTZ solution (2,4,6-tri[2'-pyridyl]-1,3,5-triazine previously prepared in 40 mM HCl), 1.25 ml of a 20 mM FeCl_3_ aqueous solution, and 12.5 ml of a 0.3 M acetate buffer (pH 3.6). In a 96-well microplate, 200 µl of the FRAP solution and 15 µl of the extracts were added in each well. Absolute ethanol was used as a blank control and, after an incubation of 30 minutes at 37°C, the absorbance was measured at 630 nm. A calibration curve was prepared using Trolox at concentrations of 20, 40, 60, 80, 100, 120, and 140 µg/ml. The results were expressed as micromoles of Trolox equivalents (TE) per gram of dry weight.

**1.7 Antimicrobial activity**

For this antimicrobial assay, the following strains were used: *Bacillus cereus FT10* (laboratory collection, isolated from *Spondias tuberosa*), *Enterococcus faecalis* ATCC 29212, *Enterococcus faecium* EM3 (laboratory collection, isolated from cheese), *Escherichia coli* ATCC 29212, *Klebsiella pneumoniae* ATCC 700603, *Listeria monocytogenes* NCTC 13627 DSM 19094, *Pediococcus acidilactici* LK8 (laboratory collection, isolated from cheese), *Pseudomonas aeruginosa* ATCC 27853, *Salmonella enterica* subsp. *enterica* serotype Enteritidis NCTC 6676, *Staphylococcus aureus* NCTC 12493 WDCM 00212, *Candida albicans* ATCC 10231, *Aspergillus brasiliensis* ATCC16404, *Botrytis cinerea* FAT1252, *Penicillium roqueforti* ATCC 10110 and *Trichoderma reesei* ATCC 26921. The antimicrobial activity was evaluated using the disk diffusion method based on the CLSI methods M02, M44 and M51, with modifications [11–13]. A volume of 10 µl of extracts was applied to 5 mm sterile discs, which were then placed on Mueller-Hinton agar previously inoculated with 100 µl of a microorganism suspension adjusted to an absorbance of 0.1. For *P. acidilactici*, it was used the MRS agar (De Man, Rogosa and Sharpe), and for *L. monocytogenes*, it was used the BHI agar (brain heart infusion). The plates were incubated at 35°C for 24 hours for bacterial and yeast strains, or at 28°C for 96 hours for mold strains. The assay was conducted in triplicate using a 60% (v/v) ethanol aqueous solution as negative control, and the diameter of the inhibition halos was measured in millimeters.

**1.8 Statistical analyses**

Statistical analyses were carried out using GraphPad Prism 8 software (GraphPad Software, San Diego, USA). To assess the differences among the groups, it was employed Student’s t-test for pairwise comparisons and one-way ANOVA for evaluating overall group variations (significance level at 5%). Following the ANOVA, Tukey’s post-hoc test was applied to identify specific group differences.

**2. References**

1. Zenebon O, Pascuet NS, Tigela P (2008) Métodos físico-químicos para análise de alimentos, 4th ed. Instituto Adolfo Lutz, São Paulo

2. AOAC (1996) Method 900.02. In: Official Methods of analysis of the Association of Official Analytical Chemists, 16th ed. Association of Official Analytical Chemists, Arlington, p 3

3. AOAC (1995) Method 920.39.C. In: Official methods of analysis of the Association of Official Analytical Chemists, 16th ed. Association of Official Analytical Chemists, Arlington, pp 10–12

4. AOAC (1995) Method 991.20. In: Official methods of analysis of the Association of Official Analytical Chemists, 16th ed. Association of Official Analytical Chemists, Arlington, pp 10–12

5. Souza GB, Nogueira ARA, Sumi LM, Batista LAR (1999) Método alternativo para a determinação de fibra em detergente neutro e detergente ácido. Embrapa Pecuária Sudeste, São Carlos

6. Kumari P, Kumari C, Singh PS (2017) Phytochemical Screening of Selected Medicinal Plants for Secondary Metabolites. International Journal of Life- Sciences Scientific Research 3:1151–1157. https://doi.org/10.21276/ijlssr.2017.3.4.9

7. Singleton VL, Orthofer R, Lamuela-Raventós RM (1999) Analysis of total phenols and other oxidation substrates and antioxidants by means of folin-ciocalteu reagent. In: Methods in Enzymology. pp 152–178

8. Mrkonjić Ž, Kaplan M, Milošević S, et al (2024) Green extraction approach for isolation of bioactive compounds in wild thyme (*Thymus serpyllum* L.) herbal dust — Chemical profile, antioxidant and antimicrobial activity and comparison with conventional techniques. Plants 13:. https://doi.org/10.3390/plants13060897

9. El Baakili A, Fadil M, Guaouguaou FE, et al (2024) Unveiling the phytochemical profile, antioxidant and antibacterial activities, acute toxicity insight and analgesic effect of Retama dasycarpa stems: An unexplored endemic plant from Morocco. Heliyon 10:. https://doi.org/10.1016/j.heliyon.2024.e37429

10. Yeerong K, Sriyab S, Somwongin S, et al (2021) Skin irritation and potential antioxidant, anti-collagenase, and anti-elastase activities of edible insect extracts. Sci Rep 11:. https://doi.org/10.1038/s41598-021-02382-0

11. CLSI (2012) Performance Standards for Antimicrobial Disk Susceptibility Tests; Approved Standard - Eleventh Edition. In: CLSI document M02A11, 11th ed. Clinical and Laboratory Standards Institute, Wayne, PA, pp 1–76

12. CLSI (2004) Method for Antifungal Disk Diffusion Susceptibility Testing of Yeasts; Approved Guideline. In: CLSI document M44-A, 1st ed. Clinical and Laboratory Standards Institute, Wayne, PA, pp 1–36

13. CLSI (2010) Method for Antifungal Disk Diffusion Susceptibility Testing of Nondermatophyte Filamentous. Fungi; Approved Guideline. In: CLSI document M51-A, 1st ed. Clinical and Laboratory Standards Institute, Wayne, PA, pp 1–46
